# In vitro susceptibility of thioredoxins and glutathione to redox modification and aging-related changes in skeletal muscle

**DOI:** 10.1016/j.freeradbiomed.2012.09.031

**Published:** 2012-12-01

**Authors:** Ivan Dimauro, Timothy Pearson, Daniela Caporossi, Malcolm J. Jackson

**Affiliations:** a Department of Health Sciences, University of Rome “Foro Italico,” 00194 Rome, Italy; b Institute of Ageing and Chronic Disease, University of Liverpool, Liverpool L69 3 GA, UK

**Keywords:** Thioredoxin, Redox Western blotting, Aging, Skeletal muscle, Free radicals

## Abstract

Thioredoxins (Trx's) regulate redox signaling and are localized to various cellular compartments. Specific redox-regulated pathways for adaptation of skeletal muscle to contractions are attenuated during aging, but little is known about the roles of Trx's in regulating these pathways. This study investigated the susceptibility of Trx1 and Trx2 in skeletal muscle to oxidation and reduction in vitro and the effects of aging and contractions on Trx1, Trx2, and thioredoxin reductase (TrxR) 1 and 2 contents and nuclear and cytosolic Trx1 and mitochondrial Trx2 redox potentials in vivo. The proportions of cytosolic and nuclear Trx1 and mitochondrial Trx2 in the oxidized or reduced forms were analyzed using redox Western blotting. In myotubes, the mean redox potentials were nuclear Trx1, −251 mV; cytosolic Trx1, −242 mV; mitochondrial Trx2, −346 mV, data supporting the occurrence of differing redox potentials between cell compartments. Exogenous treatment of myoblasts and myotubes with hydrogen peroxide or dithiothreitol modified glutathione redox status and nuclear and cytosolic Trx1, but mitochondrial Trx2 was unchanged. Tibialis anterior muscles from young and old mice were exposed to isometric muscle contractions in vivo. Aging increased muscle contents of Trx1, Trx2, and TrxR2, but neither aging nor endogenous ROS generated during contractions modified Trx redox potentials, although oxidation of glutathione and other thiols occurred. We conclude that glutathione redox couples in skeletal muscle are more susceptible to oxidation than Trx and that Trx proteins are upregulated during aging, but do not appear to modulate redox-regulated adaptations to contractions that fail during aging.

## Graphical abstract

**Graphical Abstract:**

**Figure:**
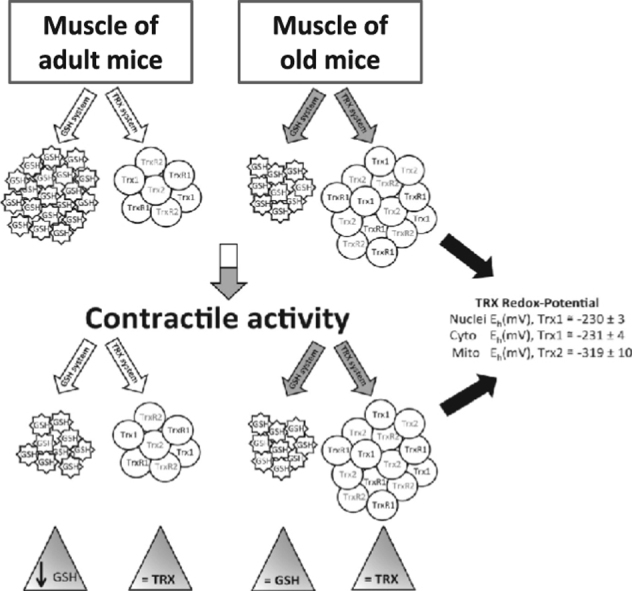

**Highlights:**

► Muscle Trx1 and Trx2 redox potentials vary between nucleus, cytosol, and mitochondria. ► Oxidants modified nuclear and cytosolic Trx1 and GSH but not mitochondrial Trx2. ► Aging increased muscle Trx contents, but did not affect Trx redox potentials. ► Contractile activity affected muscle GSH but not Trx content or redox potentials. ► Glutathione in muscle is more susceptible to physiological oxidation than Trx's.

The ability of cells and tissues from old mammals to respond to an oxidative stress by increasing the expression and activities of antioxidant defense enzymes seems severely attenuated. Previous data indicate that skeletal muscle from old rodents and humans shows a number of changes in reactive oxygen species (ROS) activities and actions in comparison to that from young rodents and humans [1]. These include increased oxidative damage to many biomolecules and a failure of ROS-stimulated (redox-regulated) signaling systems [1]. In skeletal muscle of old mice and humans there is increasing evidence that adaptations to contractile activity are attenuated [2] and many of these adaptations involve activation of redox-signaling pathways that mediate changes in muscle gene expression [3]. A feasible explanation for the inability of older muscle to respond to oxidative stress may be failed redox signaling through inappropriate oxidation of key regulatory molecules at specific subcellular sites.

Redox-dependent processes can influence the actions of many proteins involved in regulation of vital cell functions such as proliferation, differentiation, apoptosis, etc. [4,5]. Recent attention has focused on proteins that encompass thiol–disulfide regulation for which the redox status depends on a redox-active site that has an amino acid sequence containing one or two active thiols. The major intracellular thiol–disulfide systems include reduced glutathione/oxidized glutathione (GSH/GSSG) and the thioredoxin (Trx) systems. These control diverse cellular events through discrete redox pathways that influence redox signaling [6], are responsive to oxidative stress, and help maintain intracellular redox homeostasis [4]. Trx's are a class of small multifunctional proteins that are present in all prokaryotic and eukaryotic organisms and are characterized by the invariant redox active-site sequence (Trp-Cys-Gly-Pro-Cys-Lys) [5]. The oxidized (inactive) form of Trx (TrxS_2_) has two cysteines at its active site forming a disulfide bond that is reduced by thioredoxin reductases (TrxR's) in the cytosol (TrxR1) and mitochondria (TrxR2) and NADPH to a dithiol (Trx(SH)_2_), which can then act as a general protein disulfide reductase [5,7].

The Trx system is present in various cellular compartments; the Trx1 isoform is found in the nucleus and cytosol. Distinct pools are evident, as Trx1 can be imported into the nucleus from the cytoplasm during various forms of oxidative stress [8,9]. Trx2 is localized in mitochondria and is encoded by a nuclear gene and localized to the mitochondrial matrix by a mitochondrial leader sequence [10]. Trx2 is expressed ubiquitously and found at high levels in metabolically active tissue such as heart, skeletal muscle, brain, and liver, where it regulates the mitochondrial redox state and may play an important role in cell proliferation [10–12]. Homozygous knockout of either isoform of Trx in mice is embryonically lethal [13,14] and Trx molecules are conserved throughout evolution [15]. Trx1 and Trx2 interact with cysteine residues in many proteins and have been implicated in cellular processes including protein structure/folding, reductive and metabolic enzymes, energy utilization, transcription factors, and immune modulation [16]. Trx's have also been implicated in aging, and overexpression of either Trx1 or Trx2 has been shown to influence life span in experimental models [17,18].

Understanding the redox modifications to proteins within various cellular compartments such as cytosol, mitochondria, and nuclei is of importance because cell signaling events can occur in discrete compartments and antioxidant systems are not distributed uniformly throughout the cell. The development of models to understand the redox potentials in various cell compartments and how they are modified under physiological and pathophysiological conditions could help facilitate the development of novel targeted therapeutic interventions.

Thioredoxins seem to be key intracellular regulators of redox signaling and the purpose of this study was to determine the susceptibility of thioredoxins to reduction or oxidation by a physiologically relevant oxidant in various cellular compartments of a well-characterized muscle cell line in culture in comparison with the glutathione system. Additionally, anterior tibialis (AT) muscles from young and old mice were exposed to an isometric muscle contraction protocol to follow the relative changes in the redox status of Trx1 and Trx2 in various cellular compartments in comparison with their glutathione redox status. We reasoned that an increased knowledge of the relative susceptibility to oxidation/reduction of Trx1, Trx2, and GSH in various cell compartments would facilitate a greater understanding of the changes seen in muscle in response to physiological stress (contractions) or aging in vivo. To undertake this, a cell fractionation method for C2C12 myoblasts and myotubes and skeletal muscle tissue from young and old mice was initially developed and fractions were used to analyze the redox status of Trx1 (in nuclear and cytosolic fractions) and Trx2 from the same cells or tissue fraction using redox Western blotting. These cell and tissue samples were also analyzed for the protein content of Trx1, Trx2, TrxR1, and TrxR2; total protein thiol content; and glutathione and oxidized glutathione content. Data indicate that in skeletal muscle, exogenous ROS modulators modified the redox status of the Trx1 system in nuclei and cytosol; in vivo aging influenced the muscle content of Trx1, Trx2, and TrxR2; but neither aging per se nor the physiological generation of endogenous ROS by isometric contractions modified the redox potential of the Trx proteins, although under both of these conditions changes in glutathione and other thiols were seen.

## Material and methods

### Mice and isometric muscle contraction protocol

Experiments were performed in accordance with UK Home Office guidelines under the UK Animals (Scientific Procedures) Act 1986 and received ethical approval from the University of Liverpool Animal Welfare Committee. A total of 30 male C57Bl6 mice were used in this study, 15 young (mean age: 6 months old) and 15 old (mean age: 28 months old). Animals were maintained in a temperature-controlled environment fed on a standard laboratory chow diet ad libitum and subjected to a 12-h light–dark cycle.

Mice were anesthetized with ketamine hydrochloride (66 mg/kg body wt) and medatomidine hydrochloride (0.55 mg/kg body wt) by intraperitoneal injection, and anesthesia was maintained with additional ketamine (30 mg/kg body wt) as required. Each age cohort was split into three equal groups: (i) control mice, which did not receive the contraction protocol (unstimulated); (ii) mice that received the contraction protocol and were sacrificed immediately at the end of the contractions; and (iii) mice that received the contraction protocol and remained at rest for 15 min before sacrifice. For the mice in groups ii and iii, both hindlimbs were subjected to a 15-min period of electrical stimulation via surface electrodes to generate isometric contractions of the hindlimb muscles. The stimulation protocol used was a square wave pulse of 0.2 ms duration at 60 V and a frequency of 100 Hz contracted every 4 s and repeated 180 times. Mice were sacrificed by administration of an overdose of anesthetic at the times described above and both AT muscles were rapidly removed; half of one muscle was snap-frozen in liquid nitrogen for use in analyses of Trx1, Trx2, TrxR1, TrxR2, glutathione, and total protein thiol contents, and the remaining AT tissue was processed immediately to obtain subcellular fractions for redox Western blotting of Trx1 and Trx2.

### Cell culture and treatments

The C2C12 mouse skeletal myoblast line was obtained from the American Type Culture Collection (CRL-1772). C2C12 myoblasts were maintained in Dulbecco's modified Eagle's medium (DMEM; Sigma Aldrich, Poole, UK) supplemented with 1% l-glutamine (Lonza, Cologne, Germany), 10% fetal bovine serum (FBS; Biosera, Sussex, UK), and 1% penicillin and streptomycin (Sigma) under an atmosphere of 5% CO_2_ in humidified air at 37 °C. To induce myogenic differentiation, the growth medium was changed to differentiation medium (DMEM supplemented with 2% horse serum and 1% antibiotics and l-glutamine) after myoblasts had reached 90% confluence, and the cells were allowed to mature to myotubes for 7 days.

Proliferating myoblasts and differentiated myotubes were untreated, treated with hydrogen peroxide (H_2_O_2_; 300–500 μM), or treated with 5 mM dithiothreitol (DTT); the treatments were applied for 30 min at 37 °C in a tissue culture incubator. Cells were then immediately harvested for cell fractionation and biochemical analyses.

### Subcellular fractionation

Cells and freshly harvested AT tissue were homogenized in the presence of STM buffer (250 mM sucrose, 50 mM Tris, 5 mM MgCl_2_) with protease inhibitors (Sigma–Aldrich). For redox Western blots, 50 mM iodoacetic acid (IAA) was added to the lysis buffer and the pH was adjusted to pH 7.4 using 10 M NaOH (in preliminary experiments, this step was found to prevent artifactual oxidation of the Trx1 redox couple in the nuclear and cytosolic extracts). The suspensions were incubated on ice for 5 min and then centrifuged at 12,000 g for 5 min at 4 °C; the pellet (nuclear fraction) was immediately lysed in G-lysis buffer (6 M guanidine–HCl, 50 mM Tris–HCl, pH 8.3, 3 mM EDTA, 0.5% Triton X-100, 50 mM IAA; Sigma Aldrich). Proteins in the supernatant (cytosolic fraction) were precipitated in cold 100% acetone at −20 °C for 30 min followed by centrifugation at 12,000 g, and the protein pellet was resuspended in G-lysis buffer.

### Western blotting

The quality of the cell fractionation was determined for the nuclear and cytosolic fractions (15–30 μg/fraction) by examining marker proteins: histone H3 (nuclei) and glyceraldehyde-3-phosphate dehydrogenase (GAPDH; cytosol) using monoclonal antibodies for histone H3 (Cell Signaling, Hertfordshire, UK, 1:2000) and GAPDH (Abcam, Cambridge, UK, 1:5000). Whole protein extracts from cells and AT tissue (30–50 μg) were also analyzed for the content of Trx1, Trx2, TrxR1, and TrxR2 using polyclonal anti-rabbit Trx1, Trx2, and TrxR1 and anti-goat TrxR2 (Santa Cruz Biotechnology, Santa Cruz, CA, USA, 1:500). Protein samples were separated by SDS/10–14% PAGE and transferred onto a polyvinylidene difluoride membrane (Sigma). Protein was visualized after applying specific secondary horseradish peroxidase (HRP)-conjugated antibodies and exposure to a Supersignal West Dura substrate (Pierce-Thermo, Northumberland, UK).

### Analysis of the viability of C2C12 cells

Myoblasts and myotubes were tested for their sensitivity to hydrogen peroxide treatment. Cells (3×10^3^ per well) were seeded in 96-well plates and grown overnight in a tissue culture incubator in DMEM containing 10% FBS. Myoblasts were washed twice with phosphate-buffered saline (PBS) and treated with H_2_O_2_ (300 μM) for 30 min. C2C12 myotubes were allowed to differentiate for 7 days and then treated with 500 μM H_2_O_2_. Cell viability and growth were determined using a Cell Titer 96 AQ_ueous_ One Solution Cell Proliferation Assay Kit (Promega, Milan, Italy).

For analysis of apoptotic markers, 20 μg of protein from each cell extract was separated by SDS/PAGE. The following antibodies were used: anti-rabbit caspase-9 and anti-rabbit Bax (Santa Cruz Biotechnology, 1:1000) and anti-rabbit caspase-3 and anti-rabbit Bcl-2 (Cell Signaling, Milan, Italy, 1:1000).

### Redox western blotting

To allow separation of oxidized and reduced forms of Trx1 (i.e., the Trx1 redox state) homogenates were treated with IAA to stabilize the redox state by binding to reduced Trx1 and preventing further formation of oxidized Trx1. Reduced Trx1 bound to thiol-reactive IAA has a different electrophoretic mobility compared with oxidized Trx1 [19]. Briefly, after 30 min at 37 °C, excess IAA was removed using a protein desalting spin column (Pierce), and the concentration of protein in the eluate was determined by BCA protein assay (Sigma–Aldrich). Equivalent amounts of protein were loaded from each cell fraction (50–150 μg) and the reduced and oxidized Trx1 were separated by native PAGE. The proteins were electroblotted onto a nitrocellulose membrane and probed with anti-Trx1 antibody (1:2000; Abcam) followed by an HRP-conjugated secondary antibody (Cell Signaling). Bands corresponding to Trx1 were visualized using ChemiDoc XRS (Bio-Rad, Hertfordshire, UK). Bands were quantified using ImageJ software (W.S. Rasband, ImageJ, U.S. National Institutes of Health, USA). The relative contents of oxidized and reduced Trx1 were entered into the Nernst equation to calculate *E*_h_ (redox potential) values with *E*′_0_ (midpoint potential)=−240 mV at pH 7.0 at 25 °C [18].

Determination of the proportions of Trx2 in the reduced and oxidized forms (i.e., Trx2 redox state) was undertaken using the method of Go and Jones [20]. Cells were scraped into ice-cold PBS and precipitated on ice with 10% trichloroacetic acid (TCA; Fisher Scientific) for 30 min. AT tissue was minced with scissors and homogenized with an electric Potter S homogenizer (Sartorius, Surrey, UK) in PBS and then precipitated in 10% TCA for 30 min on ice. Samples were centrifuged at 12,000 g for 10 min, resuspended in 100% acetone, and incubated for 30 min on ice. After centrifugation at 12,000 g for 10 min, acetone was removed, and protein pellets were dissolved in lysis/derivatization buffer (20 mM Tris–HCl, pH 8, 15 mM 4-acetoamido-4′-maleimidylstilbene-2,2′-disulfonic acid; Molecular Probes, Paisley, UK) and incubated at room temperature (22 °C) in the dark for 3 h. The reduced and oxidized Trx2 were separated on an SDS/15% polyacrylamide gel (Geneflow, Staffordshire, UK) in the presence of nonreducing loading buffer. Immunoblotting was performed as described above, but utilized an anti-Trx2 (1:1000; Abcam) primary antibody followed by an HRP-conjugated secondary antibody. The relative contents of oxidized and reduced Trx2 were entered into the Nernst equation to calculate *E*_h_ values at 25 °C with *E*′_0_=−330 mV at pH 7.6 (mitochondrial pH).

### Preparation of samples for analysis of glutathione and total protein thiol content

An aliquot of each tissue or cell sample was mixed with 1% (w/v) sulfosalicylic acid (Sigma–Aldrich). The samples were then homogenized and centrifuged at 14,000*g* for 10 min at 4 °C, supernatants were used for the analysis of glutathione (total and oxidized forms), and the precipitated protein pellet was used for the analysis of total protein and thiol content. The protein content of samples was determined by the Bradford method.

### Glutathione and total protein thiol analyses

The total protein thiol content was analyzed by the method described by Di Monte et al. [21] adapted for use on a 96-well plate reader. The automated glutathione recycling method [22] adapted for a 96-well plate reader (Benchmark; Bio-Rad) was used to analyze the total glutathione content of samples. The oxidized glutathione content was analyzed using the same method as described for total glutathione utilizing 2-vinylpyridine [23] to prevent GSH oxidation during sample preparation. The redox states (*E*_h_) of the GSH/GSSG pools were calculated using the Nernst equation [24]. The standard midpoint potential *E*_0_ for the GSH/GSSG couple was −240 mV at pH 7.0 [25].

### Statistical analysis

Data are presented as means±SE for each experiment; for tissue and cells *n* represents the number of mice used and different day replicates, respectively. Data were evaluated for statistical significance using SPSS (version 17) using one-way ANOVA or Student's *t* test as appropriate. *P*<0.05 was considered significant.

## Results

### Efficiency of cell and tissue fractionation

Analyses of the marker proteins showed that relatively pure separation of the nuclear and cytosolic fractions was achieved for myoblasts, myotubes, and AT muscles (see Supplementary Fig. S1) using the method described.

### Analysis of the thioredoxin system in proliferating and differentiated C2C12 cells

**Fig. 1:**
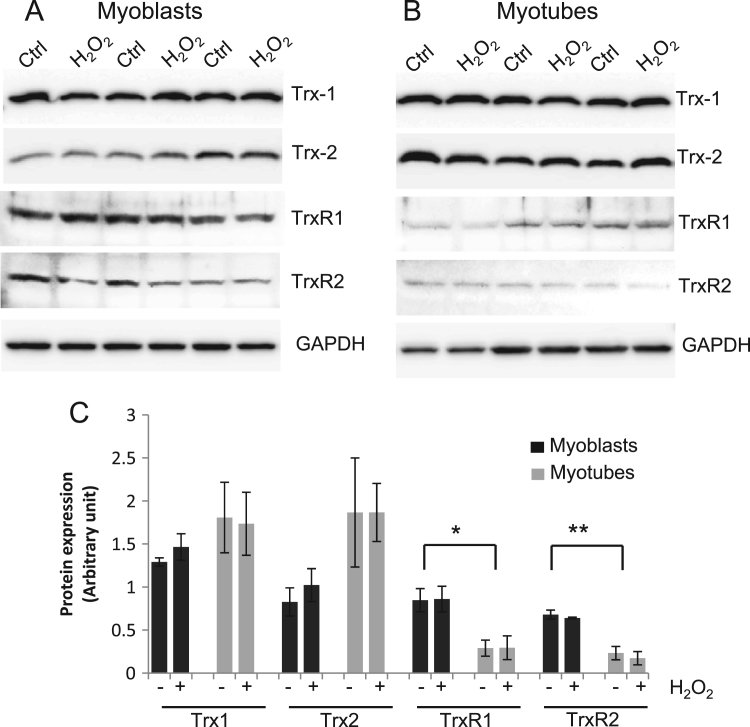
Western blot analysis of the protein contents of Trx1, Trx2, TrxR1, and TrxR2 in C2C12 myoblasts and myotubes with and without exposure to H_2_O_2_. (A) Example Western blots from myoblasts. (B) Example Western blots from myotubes. (C) Quantification of the densitometry data from Western blots showing the effect of H_2_O_2_ (+) on Trx1, Trx2, TrxR1, and TrxR2. Values are presented as means±SE. *P<0.05 cf. myoblasts, **P<0.01 cf. myoblasts.

Fig. 1 shows the relative contents of Trx1, Trx2, TrxR1, and TrxR2 obtained by Western analyses of untreated myoblasts and myotubes, together with the lack of effect of short-term exposure of myoblasts and myotubes to H_2_O_2_. Preliminary data indicated that the concentrations of H_2_O_2_ utilized had no effect on the viability nor did they induce markers of apoptosis in myoblasts or myotubes (see Supplementary Fig, S2). The contents of TrxR1 and TrxR2 in myoblasts were significantly increased compared with differentiated cells (myoblasts vs myotubes: TrxR1, 0.85±0.13 vs 0.29±0.09, *P*=0.03; TrxR2, 0.68±0.05 vs 0.23±0.08, *P*=0.008). Treatment of myoblasts or myotubes with H_2_O_2_ did not result in any changes in the content of Trx1, Trx2, TrxR1, or TrxR2 over the time course of the study.

### The sensitivity of thioredoxins to oxidation/reduction in proliferating and differentiated C2C12 cells

#### Proliferating myoblasts

**Fig. 2:**
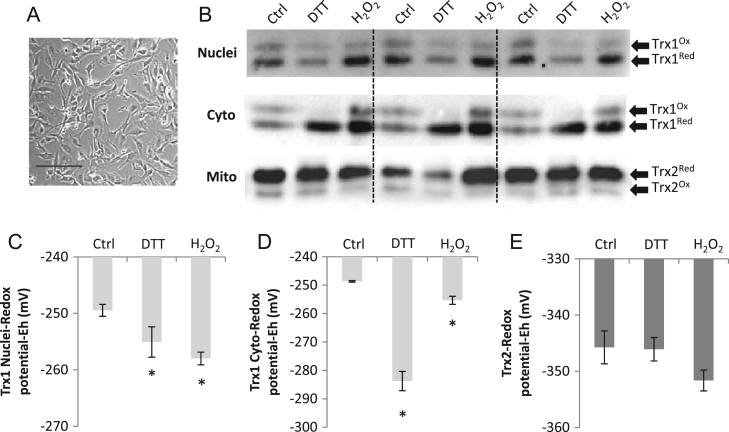
Analysis of the redox status of Trx1 in nuclear and cytoplasmic fractions and mitochondrial Trx2 in C2C12 myoblasts. (A) Light microscopy image of C2C12 myoblast cells (bar, 200 μm). (B) Example redox blots showing the redox status of Trx1 and Trx2 in untreated cells and after DTT (5 mM) or H_2_O_2_ (300 μM) treatment. Histograms showing changes in *E*_h_ values in (C) nuclear Trx1, (D) cytoplasmic Trx1, and (E) mitochondrial Trx2. The proportions of Trx1 and Trx2 in the reduced and oxidized forms were quantified by densitometry and values were entered into the Nernst equation (see Material and methods) to calculate redox potentials. Results are presented from at least three separate experiments. *P<0.05 compared with control.

The effect of treatment of C2C12 myoblasts with DTT or H_2_O_2_ on the redox status of nuclear and cytosolic Trx1 and mitochondrial Trx2 is shown in Fig. 2. The Trx1 redox blot shows two prominent immunoreactive bands in which the dominant band was fully carboxymethylated (i.e., reduced), with a less prominent band that corresponded to a partially carboxymethylated (i.e., oxidized) state. The steady-state redox potential (*E*_h_) was calculated from densitometry of the two Trx bands. In untreated control cells, Trx1 was predominantly in the reduced state in both the cytosolic and the nuclear fractions and the Trx1 redox potential in myoblasts was calculated as approximately −250 mV for both subcellular compartments (Figs. 2C and 2D). After exposure to H_2_O_2_, the myoblast Trx1 redox potential was significantly more reduced in both compartments than before treatment (control vs H_2_O_2_: nuclei, −249±0.6 mV vs −258±0.7 mV; cytoplasm, −248.7±0.3 mV vs −255.3±1.4  mV; all *P*<0.05). Treatment with the reducing agent DTT caused a significant reduction of both Trx1 compartments compared with untreated control myoblasts (Fig. 2C, DTT=−255±1.6 mV; Fig. 2D, DTT=−284±3.4 mV; *P*<0.05 vs control).

Trx2 exclusively localized to mitochondria, and two predominant forms of Trx2 were found in myoblasts representing the reduced and oxidized forms of the protein (Fig. 2B). The steady-state redox potential was calculated from densitometry of the two Trx2 bands. The redox potential of Trx2 in control untreated cells was calculated to be approximately −346 mV and this did not change significantly after treatment of cells with DTT or H_2_O_2_ (Fig. 2E).

#### Differentiated myotubes

**Fig. 3:**
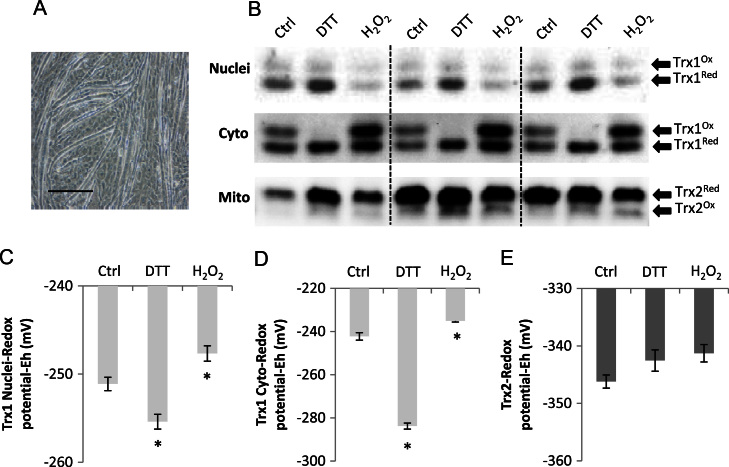
Analysis of the redox status of Trx1 in nuclear and cytoplasmic fractions and mitochondrial Trx2 in C2C12 myotubes. (A) Light microscopy image of C2C12 myotubes (bar, 200 μm). (B) Example redox blots showing the redox status of Trx1 and Trx2 in untreated myotubes and after DTT (5 mM) or H_2_O_2_ (500 μM) treatment. Histograms showing changes in *E*_h_ values in (C) nuclear Trx1, (D) cytoplasmic Trx1, and (E) mitochondrial Trx2. Results are presented from at least three separate experiments. *P<0.05 compared with control.

The effect of treatment of differentiated myotubes with DTT or H_2_O_2_ on the redox status of nuclear and cytosolic Trx1 and mitochondrial Trx2 is shown in Fig. 3. The Trx1 redox blot again showed two prominent immunoreactive bands that corresponded to the oxidized and reduced forms. In untreated myotubes, Trx1 was predominantly in the reduced state in both the cytosolic and the nuclear fractions and the Trx1 redox potential was calculated as approximately −250 mV for both subcellular compartments (Figs. 3C and 3D). After exposure to H_2_O_2_, myotube Trx1 became more oxidized in both the nuclei and the cytosol (control vs H_2_O_2_: nuclei, −251.1±0.4 mV vs −247.7±0.5 mV; cytosol, −242.2±1.7 mV vs −235.1±0.5 mV; *P*<0.05). Treatment with the reducing agent DTT caused a significant reduction of both Trx1 compartments compared with untreated control myotubes (Fig., 3C, DTT=−255.4±0.5 mV; Fig. 3D, DTT=−284±1.4 mV; *P*<0.05 vs control).

There was no change in mitochondrial Trx2 redox status in myotubes after either treatment (Fig. 3E); the redox potential of Trx2 in myotubes was approximately −346 mV. Although no differences were found between the nuclear redox potentials of Trx1 or of mitochondrial Trx2 comparing untreated myoblasts to myotubes, the cytosolic redox potential of Trx1 was significantly more oxidized in untreated myoblasts than found in untreated myotubes (*P*=0.02).

### The sensitivity of glutathione to oxidation/reduction in proliferating and differentiated C2C12 cells

#### Proliferating myoblasts

**Fig. 4:**
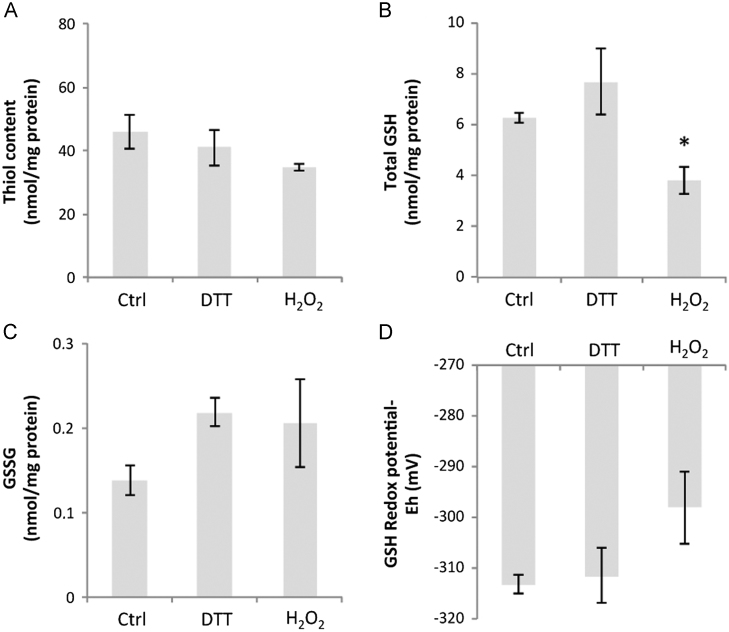
C2C12 myoblasts were untreated or exposed to either 5 mM DTT or 300 μM H_2_O_2_ for 30 min, and the (A) total thiol content, (B) total GSH, (C) GSSG, and (D) glutathione redox potential were determined. Results are presented as means±SE (*n*=3). *P<0.05 compared with control.

Total cellular protein thiol, GSH, and GSSG contents and the cellular glutathione redox potential were measured in cells treated in the same manner as for the Trx1 and Trx2 studies. Myoblast total protein thiol content was unchanged after treatment with DTT or H_2_O_2_ (Fig. 4A), the intracellular content of GSH decreased significantly with H_2_O_2_ treatment (Fig. 4B), but was unchanged by DTT treatment, whereas the myoblast content of GSSG was unchanged by either treatment as was the glutathione redox potential (Figs. 4C and 4D).

#### Differentiated myotubes

**Fig. 5:**
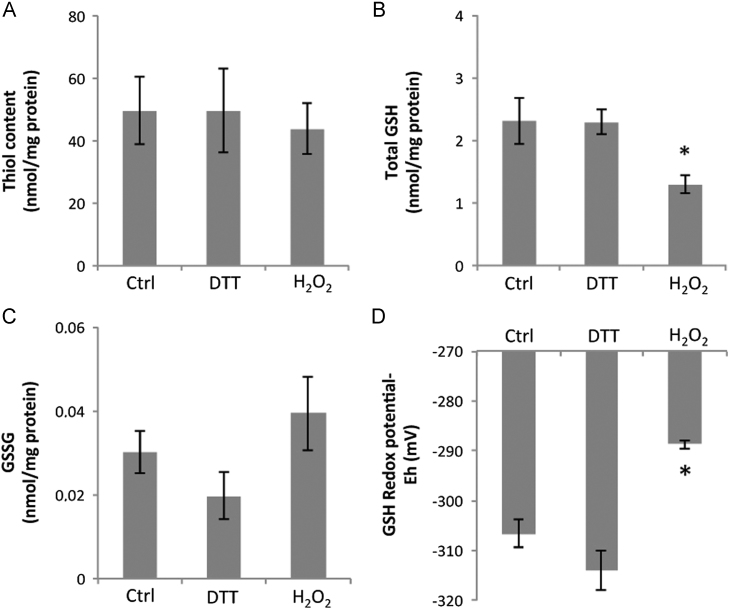
C2C12 myotubes were untreated or exposed to either 5 mM DTT or 500 μM H_2_O_2_ for 30 min, and the (A) total thiol content, (B) total GSH, (C) GSSG, and (D) glutathione redox potential were determined. Results are presented as means±SE (*n*=3). *P<0.05 compared with control and DTT-treated.

Myotube total protein thiol content was unchanged after treatment with DTT or H_2_O_2_ (Fig., 5A), the intracellular content of GSH decreased significantly with H_2_O_2_ treatment (Fig., 5B), but was unchanged by DTT treatment. The myotube GSSG content was unchanged by either treatment. There was a significant oxidation of the glutathione redox potential in response to H_2_O_2_ treatment (*P*<0.009) compared with untreated myotubes (Fig. 5D).

The content of GSH was significantly higher in myoblasts than in myotubes irrespective of treatment (myoblasts vs myotubes nmol/mg protein: control, 6.3±0.18 vs 2.3±0.32, *P*<0.05; DTT, 7.6±1.3 vs 2.3±0.17, *P*<0.05; H_2_O_2_, 3.7±0.5 vs 1.3±0.1, *P*<0.05; Fig. 4B versus 5B). The myoblast GSSG content was also up to fourfold greater than found in equivalently treated myotubes (Figs. 4C and 5C) (myoblasts vs myotubes nmol/mg protein: control, 0.13±0.02. 0.03±0.01, *P*<0.05; DTT, 0.22±0.02 vs 0.02±0.01, *P*<0.05; H_2_O_2_, 0.21±0.12 vs 0.04±0.01, *P*>0.05). No differences in GSH redox potential were found comparing myoblast to myotubes exposed to the same treatment.

### Trx1, Trx2, TrxR1, and TrxR2 content and subcellular thioredoxin pools in AT muscles from mice subjected to an isometric muscle contraction protocol

**Fig. 6:**
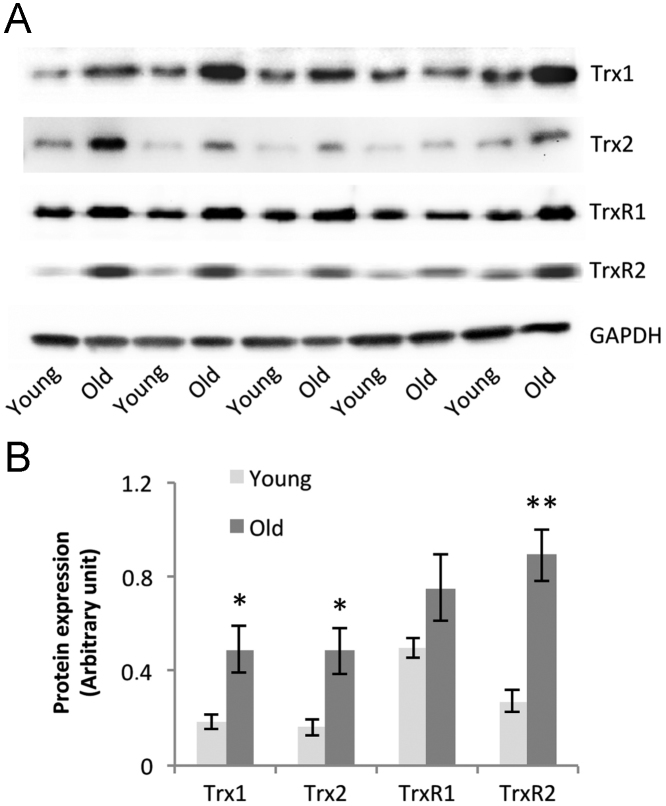
Western blot analysis of the protein content of Trx1, Trx2, TrxR1, and TrxR2 in AT muscles from young and old mice. (A) Example Western blots for Trx1, Trx2, TrxR1, and TrxR2. (B) Quantification of the densitometry data from Western blots. Values are presented as means±SE. *P<0.05 cf. values for young mice, **P<0.01 cf. values for young mice.

**Fig. 7:**
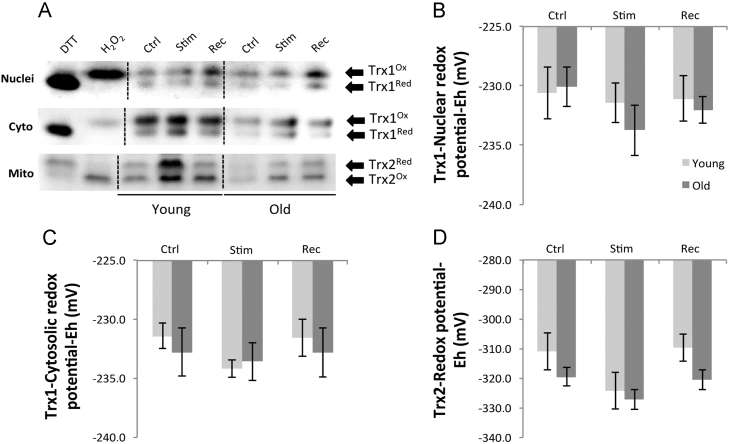
Example redox blots of nuclear and cytoplasmic Trx1 and mitochondrial Trx2 from AT muscles of young and old mice at rest (control), immediately after a 15-min isometric muscle contraction (Stim), or after a 15-min recovery period (Rec). Histograms showing changes in *E*_h_ values of (B) nuclear Trx1, (C) cytoplasmic Trx1, and (D) mitochondrial Trx2. The proportion of Trx1 and Trx2 in the reduced and oxidized forms was quantified by densitometry and values were entered into the Nernst equation (see Material and methods) to calculate redox potentials (*n*=5 animals for each group). Cells exposed to DTT and H_2_O_2_ shown in (A) were used as positive controls.

Muscles from old mice showed a significant increase in the protein contents of Trx1, Trx2, and TrxR2 compared with muscles from young mice (Fig. 6). Redox blotting of Trx1 and Trx2 showed that the muscle contraction protocol in vivo induced no significant changes in Trx1 or Trx2 oxidation status (Fig. 7), neither were the redox potentials of Trx1 and Trx2 influenced by the age of the mice. The nuclear and cytosolic Trx1 redox potential was maintained between −230 and −234 mV, whereas the mitochondrial Trx2 redox potential was consistently more reduced with values of approximately −310 mV in the resting state.

### Total protein thiol, glutathione, and oxidized glutathione contents and cellular glutathione redox potential in AT muscles from mice subjected to an isometric muscle contraction protocol

**Fig. 8:**
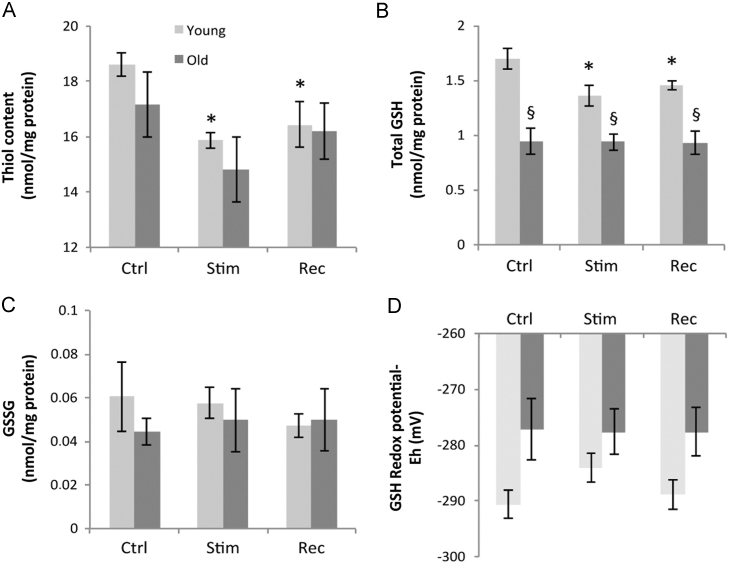
Effects of the isometric muscle contraction protocol on (A) total thiol content, (B) total GSH, (C) GSSG, and (D) glutathione redox potential of AT muscle from groups of young and old mice (*n*=5 animals for each group). *P<0.05 compared to mice in the precontractions young group. ^§^*P*<0.05 compared with values from young mice in the same pre- or postcontraction group.

The total protein thiol content of AT muscles was not altered by the age of the mice, but was significantly decreased in samples obtained immediately after contractions and at 15 min post-contractile activity in muscles from young mice (Fig. 8A). Muscles from old mice showed the same pattern of changes, but data did not reach statistical significance. The glutathione content of AT muscles from old mice was unresponsive to treatment and was significantly lower than that in muscles from young mice (Fig. 8B; young, 1.69±0.1 nmol/mg protein; old, 0.94±0.12 nmol/mg protein, *P*<0.05). The contraction protocol induced a reduction in the glutathione content of the AT muscle of young mice, which was seen both immediately after contraction and 15 min post-contractile activity (Fig. 8B; control, 1.69±0.1 nmol/mg protein; stim, 1.35±0.1; after 15 min rec, 1.45±0.04), compared with precontraction values. The GSSG contents were not different between the AT muscles from young and old mice and no effect of the contraction protocol on GSSG content was seen in either age group (Fig. 8C). The glutathione redox potential of muscles did not differ significantly between young and old mice although there was a trend toward a more oxidized redox potential in muscles from old mice, irrespective of treatment (Fig. 8D).

## Discussion

Protein oxidation with the formation of carbonyl groups and loss of free thiol groups has generally been thought to be deleterious to cells with, for example, enzyme function affected by oxidation of essential thiol groups in active sites [26], but there is growing interest in the roles of reversible oxidation of cysteine residues as a means of redox regulation of normal biological processes. The transient oxidation of thiol groups within specific proteins is the basis of redox signaling in cells and seems to be part of the processes by which cells respond to oxidants. GSH and Trx systems are key regulators of redox-sensitive pathways and also contribute to the antioxidant defenses of cells to protect cells from oxidative stress [27,28].

It has been shown that the redox state of Trx in specific cell compartments is modulated by a variety of intra- and extracellular stresses, including oxidative stress, caloric restriction, and UV, in cell lines such as human colonic epithelial cells (HT29) [29], human monocytic leukemia cells (THP1) [8,30], human keratinocytes [31], and bovine aorta endothelial cells [32]. Because no data were available for skeletal muscle cells, we utilized H_2_O_2_ and DTT to examine the effects of exogenous stresses on the redox potential of cytosolic, nuclear, and mitochondrial Trx systems using the C2C12 skeletal muscle cell line. We reasoned that understanding the susceptibility of Trx1, Trx2, and GSH to oxidation and reduction in a relevant muscle cell line would help the interpretation of changes in skeletal muscle in vivo because no previous data are available for this tissue. During contractile activity of skeletal muscle the tissue generated specific ROS and muscle cells released ROS including H_2_O_2_ [2]. The magnitude of the contraction-induced increase in H_2_O_2_ that occurs has not been well defined, but our previous calculations [33] indicate that the mean rise in intracellular H_2_O_2_ content may be less than 1 μM. Preliminary experiments undertaken for this study identified a concentration of H_2_O_2_ that modified the redox potential of Trx1 without any significant effect on cell death (see Supplementary Fig. S2). These studies (not reported in detail) identified various concentrations of H_2_O_2_ for myoblasts (300 μM) and myotubes (500 μM). Previous data also suggest that these concentrations of extracellular H_2_O_2_ will lead to a transient increase in intracellular H_2_O_2_ of 30–50 μM [33]. Although this is much higher than the mean rise in intracellular H_2_O_2_ calculated to occur during contractile activity, there is no current information on local concentrations of H_2_O_2_ that occur adjacent to the sites of generation although these must inevitably be considerably higher. Several previous studies have investigated the susceptibility of myoblasts and myotubes to oxidation induced by H_2_O_2_ but there is still debate about their relative susceptibilities [34,35].

Halvey et al. [31] and Hansen et al. [36] have reported that the Trx systems in various cell compartments are not in redox equilibrium and that typically mitochondria are most reduced, nuclei are intermediate, and the cytoplasm is most oxidized. This pattern was observed in this study in proliferating and differentiated C2C12 cells and in intact AT muscles. In untreated cells, the nuclear, cytosolic, and mitochondrial pools of Trx's were predominantly in the fully reduced form; nuclear Trx1 was ∼70% reduced, cytosolic Trx1 was ∼60% reduced, and mitochondrial Trx2 was ∼80% reduced. The calculated redox potentials of the various pools of dithiols were nuclear Trx1 approximately −250 mV, cytosolic Trx1 approximately −240 mV, and Trx2 approximately −345 mV.

Rohrbach et al. [37] reported a modification of components of the thioredoxin system after oxidative stress, but application of H_2_O_2_ to C2C12 myoblasts over the time course of this study did not modify the content of Trx1, Trx2, TrxR1, or TrxR2, but induced an unexpected decrease in the redox potential of Trx1 (i.e., this became more reduced). The mechanisms underlying these findings are unclear, but our data do not support the possibility that this was due to modification of TrxR content. A modification of cell NADPH content might explain the changes observed, but unfortunately it was not possible to study this aspect as part of this study.

Similarly, treatment with H_2_O_2_ did not change the protein content of the components of the thioredoxin system in differentiated C2C12 myotubes, although we observed that the basal levels of TrxR1 and TrxR2 were significantly lower than found in myoblasts as was reported previously [38]. Results from differentiated myotubes also provided evidence that oxidation of individual subcellular compartments can occur in the absence of a generalized cellular oxidation. C2C12 myotubes showed oxidation of cytosolic and nuclear Trx1 without affecting the redox state of Trx2. Trx1 and Trx2 belong to the same protein family and both contain conserved active centers, which show similar in vitro oxidation by ROS and function as critical components of the local antioxidant network in specific subcellular compartments. Several studies have demonstrated they have different sensitivities to an external oxidative stress [10,30,31]. These data suggest that Trx2 was less prone to oxidation in vitro and that the Trx2 redox state was regulated independent of that of the Trx1 pool. Trx1 was found to respond to exogenous H_2_O_2_, whereas mitochondrial Trx2 was unaffected. The physical structure of muscle cells may play a role in influencing access to subcellular organelles, as may the relative differences in the volumes of the mitochondrial reticulum within myotubes and myoblasts. To further characterize the response of Trx's to oxidation/reduction, myoblasts and myotubes were treated with DTT, a commonly used agent to reduce disulfide bonds in proteins [39]. The data obtained indicate that both nuclear and cytoplasmic Trx1 were more responsive to DTT treatment than mitochondrial Trx2 in a manner analogous to the pattern seen after treatment with H_2_O_2_.

In general, for both myoblasts and myotubes, the Trx1 redox potentials responded similarly to the treatments, with Trx2 in both cell types being unresponsive to treatment and successfully maintaining the reduced environment that is necessary for normal electron transport chain activity.

Analysis of the GSH content of cells showed that the concentration was higher in proliferating C2C12 cells than in differentiated cells. Previous studies have reported that GSH concentrations are highest when cells are preparing for, or actively undergoing, cell division, followed by a substantial decrease in the intracellular GSH concentration in cells that are not dividing and expressing markers of differentiation [40,41]. In this case the concentrations of GSH may relate to the cell's metabolic capacity to generate ROS through cellular processes, because proliferating myoblasts are more metabolically active than differentiated myotubes. After H_2_O_2_ treatment, both myoblasts and myotubes showed significant decreases in total GSH. However, the GSH/GSSG redox potential became significantly more oxidized only in myotubes. These data are in accord with the proposal by Jones and Go [42] that GSH is more responsive to oxidative signaling in differentiated cells than in proliferating cells.

We hypothesized that understanding the redox sensitivities of subcellular compartments in skeletal muscle might be a potentially powerful approach for identifying new pathways and pharmacological tools to influence muscle function and target antioxidant defenses particularly during aging. Trx's in vivo are exposed to diverse and dynamic microenvironments and there are no appropriate in vitro models to study these antioxidant systems within skeletal muscles during aging. The data obtained indicate that aging in skeletal muscle is associated with an increase in the muscle content of Trx1, Trx2, and TrxR2, changes that may reflect aging-related adaptations to maintain Trx function and cellular redox balance and prevent oxidative damage. Overexpression of Trx1 or Trx2 has previously been shown to influence life span in mice [17,18], supporting the key role played by this protein in aspects of the aging process.

We report here the redox potentials of Trx and GSH in subcellular compartments of the skeletal muscle system of old and young mice in vivo. We also report the relative changes in the redox status of Trx1 and Trx2 from different cellular compartments in comparison with the glutathione redox status of AT muscles of young and old mice exposed to an isometric muscle contraction protocol previously shown to induce an increase in intracellular ROS [43,44].

Current literature on the status of the Trx system during aging is scant and this area has not been studied extensively [45,46]. These results suggest that a tissue-specific modulation of Trx activity may be related to the functional state of the organ under oxidative stress. The current work has shown that the redox status of both Trx's was unchanged in muscle from old compared with young mice although there was an increase in Trx1 and Trx2 content in muscle from old mice. These data suggest that the increase in Trx1, Trx2, and TrxR2 proteins is a compensatory response to counteract increased oxidative stress during the aging process [47]. Data also support the possibility that the increase in ROS generated during the contraction protocol must be relatively modest in young and old mice and unlikely to present a major “oxidative stress” sufficient to oxidize the Trx's (despite the protocol being sufficient to cause changes in total muscle protein thiols and glutathione levels).

Published data indicate that aging is associated with a decrease in the extracellular GSH/GSSG redox potential [6], but estimates of intracellular redox potentials from muscles of old organisms have not been undertaken. In common with other studies [2,48,49], our data from skeletal muscles of old and young mice indicate that the oxidized glutathione content did not differ, but the total glutathione content of quiescent muscles from old mice was significantly lower than that of young mice although the GSH redox potentials were not significantly different comparing either the control or the recovery groups (*P*=0.07). The total protein thiol content of muscles from old mice was unchanged in the group compared with the young group and showed no significant response after contractions. Significant effects of contractile activity were seen only in the muscles of young mice in which both the total protein thiol content and the glutathione content decreased after contractions as previously reported [2].

Thus in proliferating and differentiated muscle cells and in mouse muscle in vivo, glutathione and the total cell content of protein thiols seem more prone to oxidation during physiological stimuli than either Trx1 or Trx2. Unfortunately it is not possible to measure subcellular compartments of GSH in a manner similar to Trx1 and Trx2, but the data obtained support the possibility that GSH is more sensitive to exogenous and endogenous oxidants than Trx1 or Trx2. The cause of the decrease in GSH that occurs in muscle from young mice in response to contractions is unclear but has been previously attributed to oxidation of GSH by endogenous ROS generated during contractile activity and subsequent loss of the oxidized GSSG from the muscle cell [50]. Recent data have implicated the contraction-induced changes in cellular redox potentials as a key mechanistic step in specific adaptive responses to contractions that fail in aging [3], and the current GSH and protein thiol data are fully compatible with this, but also fail to implicate changes in Trx1 or Trx2 in the process.

In conclusion, these data provide the first clear evidence that skeletal muscle aging is associated with changes in the expression of Trx and the GSH system but not with oxidation/reduction of thioredoxin proteins and that the physiological stress of contractions also predominantly influences muscle GSH rather than Trx1 or Trx2. The development of methods to measure the redox status of specific proteins in various organelles will allow more complete delineation of the role of compartment-specific redox pathways and how they are influenced by aging. Many age-related disorders are associated with changes in redox states and redox regulation of protein thiols. The development of models that establish the interconnections between redox pathways and their responses during oxidative stress will aid our understanding of the complex regulation of redox processes under normal and pathological conditions.

## Footnotes

Appendix A Supplementary data associated with this article can be found in the online version at doi:10.1016/j.freeradbiomed.2012.09.031.
