# Supplementary material for: In vitro susceptibility of thioredoxins and glutathione to redox modification and aging-related changes in skeletal muscle
Source: Free Radic Biol Med. 2012 Dec 1;53(11):2017–27. doi: 10.1016/j.freeradbiomed.2012.09.031 (PMC3657158; doi:10.1016/j.freeradbiomed.2012.09.031)
Supplement: Supplementary file 1 — Supplementary Material [file mmc1.ppt]

## Slide 1
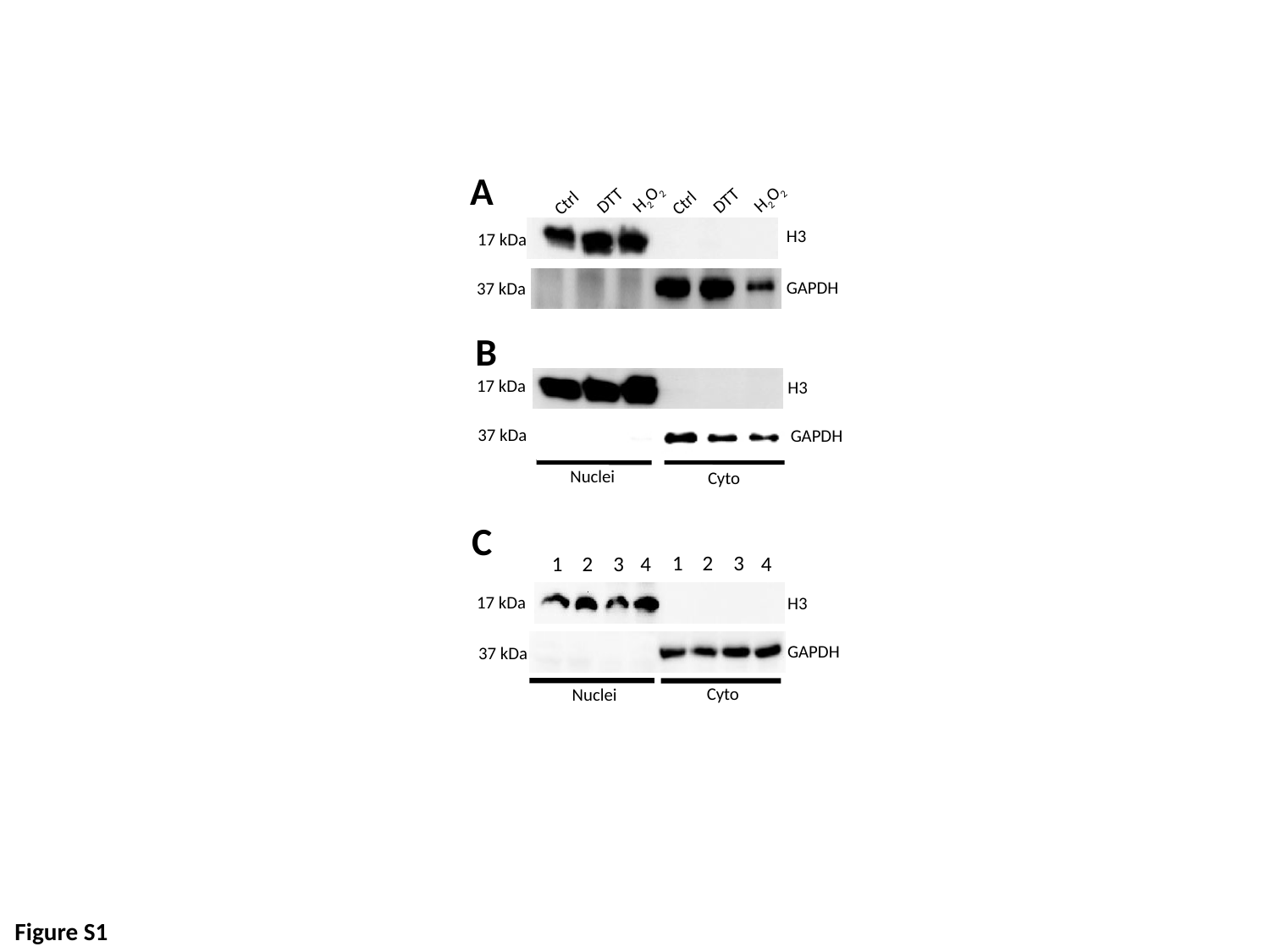

A
H2O2
H2O2
DTT
DTT
Ctrl
Ctrl
 H3
17 kDa
GAPDH
37 kDa
B
17 kDa
H3
37 kDa
GAPDH
Nuclei
Cyto
C
2
3
1
4
2
3
1
4
17 kDa
H3
GAPDH
37 kDa
Cyto
Nuclei
Figure S1

## Slide 2
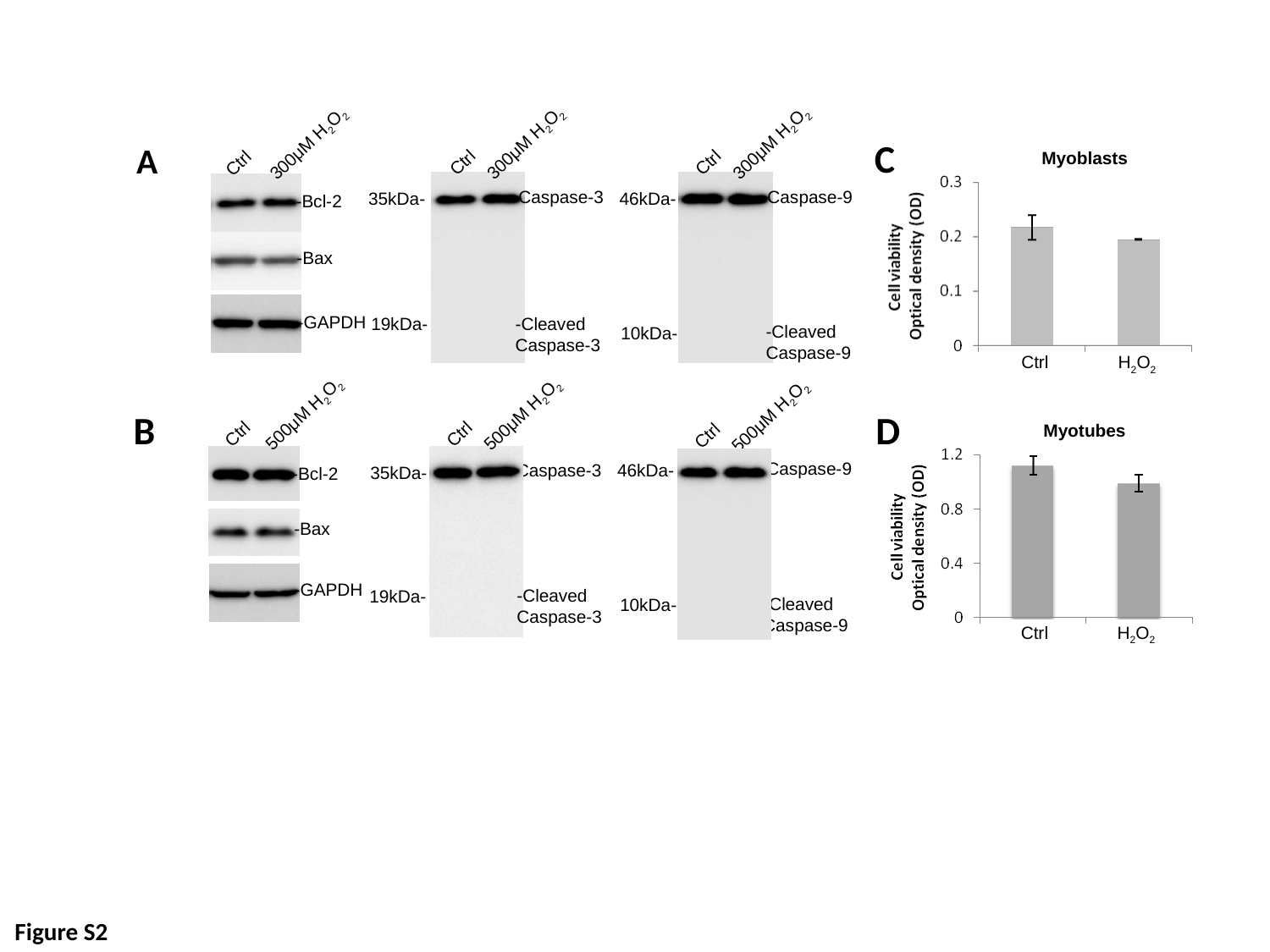

300μM H2O2
Ctrl
-Caspase-3
35kDa-
19kDa-
-Cleaved
Caspase-3
300μM H2O2
Ctrl
-Caspase-9
46kDa-
-Cleaved
Caspase-9
10kDa-
300μM H2O2
Ctrl
-Bcl-2
-Bax
-GAPDH
A
500μM H2O2
Ctrl
-Bcl-2
-Bax
-GAPDH
500μM H2O2
Ctrl
-Caspase-3
-Cleaved
Caspase-3
35kDa-
19kDa-
500μM H2O2
Ctrl
-Caspase-9
-Cleaved
Caspase-9
10kDa-
46kDa-
B
C
Myoblasts
Ctrl
H2O2
D
Myotubes
Ctrl
H2O2
Figure S2
